# Draft Genome Sequence of *Salmonella enterica* Serovar Senftenberg 070885 and Its Linalool-Adapted Mutant

**DOI:** 10.1128/genomeA.01036-17

**Published:** 2017-10-12

**Authors:** Amit Hollander, Emmanuel Kalily, Dina Shachar, Sima Yaron, Yael Danin-Poleg

**Affiliations:** Faculty of Biotechnology and Food Engineering, and the Russell Berrie Nanotechnology Institute, Technion–Israel Institute of Technology, Haifa, Israel

## Abstract

Here we report the genome sequences of both *Salmonella* Senftenberg 070885, a clinical isolate from the 2007 outbreak linked to basil, and its mutant linalool-adapted *S*. Senftenberg (LASS). These draft genomes of *S*. Senftenberg may enable the identification of bacterial genes responsible for resistance to basil oil.

## GENOME ANNOUNCEMENT

*Salmonella enterica* serovar Senftenberg (*S.* Senftenberg) was linked to an international foodborne outbreak in 2007, which originated from contaminated basil that caused more than 50 primary cases in Europe and the United States (1). *S*. Senftenberg strain 070885, a clinical isolate from this outbreak, possessed an increased resistance to basil oil and each of its major compounds, which correlated with better survival on basil plants before and after harvest (2, 3). Recent studies have shown that *Salmonella* strains can develop strategies to adapt to stressful conditions on the plant, including the presence of antimicrobial compounds (2, 3). A selective pressure of linalool, one of the major constituents of basil oil (4, 5), applied on the 070885 strain resulted in a linalool-adapted *S*. Senftenberg (LASS) strain. LASS possessed multiresistance to linalool, basil oil, and several antibiotics and demonstrated better survival on harvested basil leaves (6). Contamination of fresh produce with human pathogens, together with the emergence of pathogen resistance to natural antimicrobial agents and cross-resistance to antibiotics, have significant impacts on human health worldwide.

Here we describe the draft genome sequences of two *Salmonella enterica* subsp.* enterica* serovar Senftenberg strains, 070885 and its LASS mutant. Both strains were grown at the Technion and subjected to whole-genome shotgun sequencing.

Two libraries were prepared using Illumina-compatible Nextera DNA sample prep kits (7, 8) and sequenced using Illumina HiSeq Rapid, generating 1,553,168 and 2,426,962 pass-filtered, 150-bp paired-end reads for the 070885 and LASS strains, respectively. The trimmed reads (using k-mer values of 27) were *de novo* assembled using Geneious 10.0.9 (9). The assemblies for 070885 and LASS cover 4,816,312 bp and 4,791,611 bp, with *N*_*5*0_ values of 43,053 and 96,230 bp and longest segments of 153,977 and 278,368 bp, respectively. Genome coverages were 46×and 72× for 070885 and LASS, respectively. Mapping was done to validate the *de novo* assemblies using Geneious 10.1 (9). Ninety-three percent and 84% of the contigs of 070885 and LASS were mapped to one chromosome and three plasmids of the human isolate *S*. Senftenberg NCTC10384 genome (BioSample number SAMEA2517359, GenBank assembly accession number GCA_001457675.1).

The draft genomes of 070885 and LASS consist of 197 and 107 segments, respectively, with a minimum of 500 bp, covering 4.8 Mbp (52.1% G+C content), which is similar to the estimated genome length of the other sequenced *S*. Senftenberg strains (e.g., NCTC10384). Totals of 4,910 and 4,839 coding sequences (CDS), 218 and 187 pseudogenes, 25 and 20 rRNAs, 78 and 80 tRNAs, and 12 and 13 noncoding RNAs (ncRNAs) for 070885 and LASS, respectively, were predicted and annotated by the NCBI Prokaryotic Genome Annotation Pipeline (10), results which were similar to those of the annotation predicted by Rapid Annotations using Subsystems Technology (RAST) (11).

The whole-genome sequences of both *S*. Senftenberg 070885 and its resistant mutant LASS may lead to the discovery of specific mutations and bacterial genes which may be involved in persistence on herbs, as well as multiantibiotic resistance, and thus pose increased risks to public health.

### Accession number(s).

This whole-genome shotgun project has been deposited at DDBJ/EMBL/GenBank under the accession numbers NJPX00000000 and NITT00000000 for strains 070885 and LASS, respectively. The versions described in this paper are versions NJPX01000000 and NITT01000000.
